# The utility of PRISMA for abstracts in two cohorts: a comparison of health technology assessment and Cochrane database of systematic reviews 2009-2014

**DOI:** 10.1186/1745-6215-16-S2-P163

**Published:** 2015-11-16

**Authors:** Emma Kirkpatrick, Amanda Young, Kathy Tier, Peter Davidson

**Affiliations:** 1 National Institute for Health Research Evaluation, Trials and Studies Coordinating Centre, Southampton, UK; 2 University of Southampton, Southampton, UK

## Background

Abstracts of systematic reviews (SRs) play an important role in dissemination of results, with most evidence users consulting the abstract before reading a full article and some only ever consulting the abstract. However, abstracts of SRs are often poorly reported. The PRISMA statement (Liberati et al., 2009) for reporting SRs was extended to include guidelines for the production of abstracts (Beller et al., 2013) to improve this.

## Objectives

We aimed to conduct an exploratory evaluation of the adherence to PRISMA guidelines for SR abstracts in two cohorts: *Health Technology Assessment* (HTA) and *Cochrane Database of Systematic Reviews* (CDSR).

## Methods

SRs published in *HTA* and *CDSR* between 2009-2014 were eligible for inclusion in the study. Twenty SRs from each year were randomly selected from both cohorts and their abstracts assessed against the PRISMA for abstracts checklist (Beller et al., 2013). Two researchers working independently extracted the data; queries were explored and resolved by the team.

## Results

Results will report adherence to guidelines in both cohorts and examine areas of weakness. Impact of PRISMA guidelines will be assessed by examining improvements in reporting standards over time, in particular by comparing publications submitted before and after 2013.

## Conclusions

The PRISMA for abstracts checklist may have limited utility for complex SRs which include multiple comparisons and economic evaluations. We will discuss possible modifications to increase its usefulness, contributing to complete reporting of complex SR abstracts.

